# Supplementary material for: Patterns of Intron Gain and Loss in Fungi
Source: PLoS Biol. 2004 Nov 30;2(12):e422. doi: 10.1371/journal.pbio.0020422 (PMC532390; doi:10.1371/journal.pbio.0020422)
Supplement: Table S1 — Also available at http://genes.mit.edu/NielsenEtAl/. (4.3 MB ZIP). [file pbio.0020422.st001.zip › NielsenEtAl/html/213.html]

AN0116.1.NCU04336.1.MG06071.1.FG06204.1


```
 CLUSTAL W (1.82) Multiple Sequence Alignments - Introns Inserted


Sequence 1: NCU04336.1	158 aa
Sequence 2: FG06204.1	156 aa
Sequence 3: MG06071.1	157 aa
Sequence 4: AN0116.1	153 aa
Alignment Length: 158 aa
Number Identitical Residues: 93 aa
Alignment Score (without introns) 4345


MG06071.1 	MSKAR-AAAGGADQIVKLLVGAGQASPSPPVGPALGSKGVKSMDFCK0EFNARTAHIITG
NCU04336.1	MSKAAKGGAGGVEQVVKLIVGAGQASPSPPVGPALGSKGIKSMDFCK0EFNARTAHINTG
FG06204.1 	MSKAR--GAGGVDQIVKLIVGAGQASPSPPVGPALGSKGVKSMDFCK0EFNARTAHIITG
AN0116.1  	MAKKA----IAKDQIVKLIVGAGQASPSPPVGPALGSKGVKSMDFCK0EFNARTAHITTG
          	*:*       . :*:***:********************:******* ********* **

MG06071.1 	TPTPVRVVVRPDRTFSFDIRTPQTSWLLLNAADAPMGRKGKRKGTGLAGKETVGTVSLKH
NCU04336.1	TPMPVRVTVRPDRSFHFDVRTPHTSWLLLNAAEAPIGKGGKRKGASNPGKEVVGTVSLKH
FG06204.1 	TPTPCRVTVRPDRSFTFDVRTPQTSWLLLNAVEAPMGKKGKRKGAGKPGHETVGTITLKH
AN0116.1  	VPIPARVTVRPDRSFHFDLRTPTTTYLLLQAAKVEP-RKNRIRGAQRPGHEFIGKISLKH
          	.* * **.*****:* **:*** *::***:*...   : .: :*:  .*:* :*.::***

MG06071.1 	IYEIAKIKQTEMRLSGHSLEGLCKSIIYQCKSIGVEVKP
NCU04336.1	VYEIAKIKQSELRLSGLPLEGLCRAVIYQARSIGINVIP
FG06204.1 	VYEIAKIKQSELRLSGLSLEGLCRAVIFQAKSIGIDVVA
AN0116.1  	VYEIAKIKQSETRLSGLSLEGLCKSVMAQAKSVGIQVVP
          	:********:* **** .*****:::: *.:*:*::* .
```
